# A Duet for one^☆^

**DOI:** 10.1016/j.concog.2014.12.003

**Published:** 2015-11

**Authors:** Karl Friston, Christopher Frith

**Affiliations:** The Wellcome Trust Centre for Neuroimaging, Institute of Neurology, UCL, United Kingdom

**Keywords:** Communication, Theory of mind, Active inference, Predictive coding, Attention, Sensory attenuation, Bayesian, Generalised synchrony

## Abstract

This paper considers communication in terms of inference about the behaviour of others (and our own behaviour). It is based on the premise that our sensations are largely generated by other agents like ourselves. This means, we are trying to infer how our sensations are caused by others, while they are trying to infer our behaviour: for example, in the dialogue between two speakers. We suggest that the infinite regress induced by modelling another agent – who is modelling you – can be finessed if you both possess the same model. In other words, the sensations caused by others and oneself are generated by the same process. This leads to a view of communication based upon a narrative that is shared by agents who are exchanging sensory signals. Crucially, this narrative transcends agency – and simply involves intermittently attending to and attenuating sensory input. Attending to sensations enables the shared narrative to predict the sensations generated by another (i.e. to listen), while attenuating sensory input enables one to articulate the narrative (i.e. to speak). This produces a reciprocal exchange of sensory signals that, formally, induces a generalised synchrony between internal (neuronal) brain states generating predictions in both agents. We develop the arguments behind this perspective, using an active (Bayesian) inference framework and offer some simulations (of birdsong) as proof of principle.

## Highlights

• This paper casts theory of mind as inferring the causes of sensory signals.
• In demonstrates the emergence of neuronal synchronisation during active inference.
• It shows how attention to and attenuation of sensations enables communication.
• This provides a (mathematical) image of communication as generalised synchrony.

## 1 Introduction

One of the most intriguing issues in (social) neuroscience is how people infer the mental states and intentions of others. In this paper, we take a formal approach to this issue and consider communication in terms of mutual prediction and active inference (De Bruin & Michael, 2014; Teufel, Fletcher, & Davis, 2010). The premise behind this approach is that we model the causes of our sensorium – and adjust those models to maximise Bayesian model evidence or, equivalently, minimise surprise (Brown & Brün, 2012; Kilner, Friston, & Frith, 2007). This perspective on action and perception has broad explanatory power in several areas of cognitive neuroscience (Friston, Mattout, & Kilner, 2011) – and enjoys support from several lines of neuroanatomical and neurophysiological evidence (Egner & Summerfield, 2013; Rao & Ballard, 1999; Srinivasan, Laughlin, & Dubs, 1982). Here, we apply this framework to communication and consider what would happen if two Bayesian brains tried to predict each other. We will see that Bayesian brains do not predict each other – they predict themselves; provided those predictions are enacted. The enactment of sensory (proprioceptive) predictions is a tenet of *active inference* – under which we develop this treatment. In brief, we consider the notion that a simple form of communication emerges (through generalised synchrony) if agents adopt the same generative model of communicative behaviour.

So how do prediction and generative models speak to theory of mind? The premise here is that we need to infer – and therefore predict – the causes of sensations to perceive them. For example, to perceive a falling stone we have to appeal (implicitly) to a model of how objects move under gravitational forces. Similarly, the perception of biological motion rests on a model of how that motion is caused. This line of argument can be extended to the perception of intentions (of others or ourselves) necessary to explain sensory trajectories; particularly those produced by communicative behaviour. The essential role of inference and prediction has been considered from a number of compelling perspectives: see (Baker, Saxe, & Tenenbaum, 2009; Frank & Goodman, 2012; Goodman & Stuhlmuller, 2013; Kiley Hamlin, Ullman, Tenenbaum, Goodman, & Baker, 2013; Shafto, Goodman, & Griffiths, 2014). This Bayesian brain perspective emphasises the role of prediction in making inferences about the behaviour of others; particularly in linguistic communication. We pursue exactly the same theme; however, in the context of active inference. Active inference takes the Bayesian brain into an embodied setting and formulates action as the selective sampling of data to minimise uncertainty about their causes. This provides a natural framework within which to formulate communication, which is inherently embodied and enactivist in nature. In brief, we hope to show that active inference accounts for the circular (Bayesian) inference that is inherent in communication.

The very notion of *theory of mind* speaks directly to inference, in the sense that theories make predictions that have to be tested against (sensory) data. In what follows, we focus on the implicit model generating predictions: imagine two brains, each mandated to model the (external) states of the world causing sensory input. Now imagine that sensations can only be caused by (the action of) one brain or the other. This means that the first brain has to model the second. However, the second brain is modelling the first, which means the first brain must have a model of the second brain, which includes a model of the first – and so on *ad infinitum*. At first glance, the infinite regress appears to preclude a veridical modelling of either brain’s external states (i.e., the other brain). However, this infinite regress dissolves if the two brains are formally similar and each brain models the sensations caused by itself and the other as being generated in the same way. In other words, if there is a shared narrative or dynamic that both brains subscribe to, they can predict each other exactly, at least for short periods of time. This is basic idea that we pursue in the context of active inference and predictive coding. In fact, we will see that this solution is a necessary and emergent phenomenon, when two or more (formally similar) active inference schemes are coupled to each other. Mathematically, the result of this coupling is called *generalised synchronisation* (aka synchronisation of chaos).

Generalized synchrony refers to the synchronization of chaotic dynamics, usually in skew-product (i.e., master–slave) systems (Barreto, Josic, Morales, Sander, & So, 2003; Hunt, Ott, & Yorke, 1997). However, we will consider generalized synchrony in the context of reciprocally coupled dynamical (active inference) systems. This sort of generalized synchrony was famously observed by Huygens in his studies of pendulum clocks – that synchronized themselves through the imperceptible motion of beams from which they were suspended (Huygens, 1673). This nicely illustrates the *action at a distance* among coupled dynamical systems. Put simply, generalised synchronisation means that knowing the state of one system (e.g., neural activity in the brain) means that one can predict the states of the other (e.g., another’s brain). The sequence or trajectory of states may not necessarily look similar but there is a quintessential coupling in the sense that the dynamics of one system can be predicted from state of another. In this paper, we will illustrate the emergence of generalized synchronization when two predictive coding schemes are coupled to each other through action. In fact, we will illustrate a special case of generalized synchrony; namely, *identical synchronization*, in which there is a one-to-one map between the states of two systems. In this case, there is a high mutual predictability and the fluctuations in the states appear almost the same. In short, we offer generalized synchronization as a mathematical image of communication (of a simple sort) that enables two Bayesian brains to entrain each other and, effectively, share the same dynamical narrative.

This paper comprises five sections. The first provides a brief review of active inference and predictive coding, with a focus on the permissive role of sensory attenuation when acting on the world. In predictive coding, sensory attenuation is a special case of optimising the precision or confidence in sensory (and extrasensory) information that is thought to be encoded by the gain of neuronal populations encoding prediction errors (Clark, 2013a; Feldman & Friston, 2010). This takes us into the realm of cortical gain control and neuromodulation – that may be closely tied to synchronous gain and the oscillatory dynamics associated with binding, attention and dynamic coordination (Fries, Womelsdorf, Oostenveld, & Desimone, 2008; Womelsdorf & Fries, 2006). In the second section, we introduce a particular model that is used to illustrate perception, action and the perception of action in the context of communication. This model has been used previously to illustrate several phenomena in perception; such as perceptual learning, repetition suppression, and the recognition of stimulus streams with deep hierarchical structure (Friston & Kiebel, 2009; Kiebel, Daunizeau, & Friston, 2008). In the third section, we provide a simple illustration of omission related responses – that are ubiquitous in neurophysiology and disclose the brain’s predictive proclivity. In the fourth section, we use this model to illustrate the permissive role of sensory attenuation in enabling action by simulating a bird that sings to itself. The purpose of this section is to show that song production requires the attenuation of sensory input during singing – that would otherwise confound perceptual inference due to sensorimotor delays. This leads to the interesting (and intuitive) notion that the sensory consequences of acting have to be attenuated in order to act. This means that one can either talk or listen but not do both at the same time. Armed with this insight, we then simulate – in the final section – two birds that are singing to themselves (and each other) and examine the conditions under which generalised synchrony emerges. These simulations are offered as proof of principle that communication (i.e., a shared dynamical narrative) emerges when two dynamical systems try to predict each other.

## 2 Active inference and predictive coding

Recent advances in theoretical neuroscience have inspired a (Bayesian) paradigm shift in cognitive neuroscience. This shift is away from the brain as a passive filter of sensations towards a view of the brain as a statistical organ that generates hypotheses or fantasies (from Greek *phantastikos*, the ability to create mental images, from *phantazesthai*), which are tested against sensory evidence (Gregory, 1968). This perspective dates back to the notion of unconscious inference (Helmholtz, 1866/1962) and has been formalised in recent decades to cover deep or hierarchical Bayesian inference – about the causes of our sensations – and how these inferences induce beliefs, movement and behaviour (Clark, 2013b; Dayan, Hinton, & Neal, 1995; Friston, Kilner, & Harrison, 2006; Hohwy, 2013; Lee & Mumford, 2003).

### 2.1 Predictive coding and the Bayesian brain

Modern formulations of the Bayesian brain – such as predictive coding – are now among the most popular explanations for neuronal message passing (Clark, 2013b; Friston, 2008; Rao & Ballard, 1999; Srinivasan et al., 1982). Predictive coding is a biologically plausible process theory for which there is a considerable amount of anatomical and physiological evidence (Friston, 2008; Mumford, 1992). See (Bastos et al., 2012) for a review of canonical microcircuits and hierarchical predictive coding in perception and (Adams, Shipp, & Friston, 2012; Shipp, Adams, & Friston, 2013) for a related treatment of the motor system. In these schemes, neuronal representations in higher levels of cortical hierarchies generate predictions of representations in lower levels. These top–down predictions are compared with representations at the lower level to form a prediction error (usually associated with the activity of superficial pyramidal cells). The ensuing mismatch signal is passed back up the hierarchy, to update higher representations (associated with the activity of deep pyramidal cells). This recursive exchange of signals suppresses prediction error at each and every level to provide a hierarchical explanation for sensory inputs that enter at the lowest (sensory) level. In computational terms, neuronal activity encodes beliefs or probability distributions over states in the world that cause sensations (e.g., my visual sensations are caused by a *face*). The simplest encoding corresponds to representing the belief with the expected value or *expectation* of a (hidden) cause. These causes are referred to as *hidden* because they have to be inferred from their sensory consequences.

**Fig. 1:**
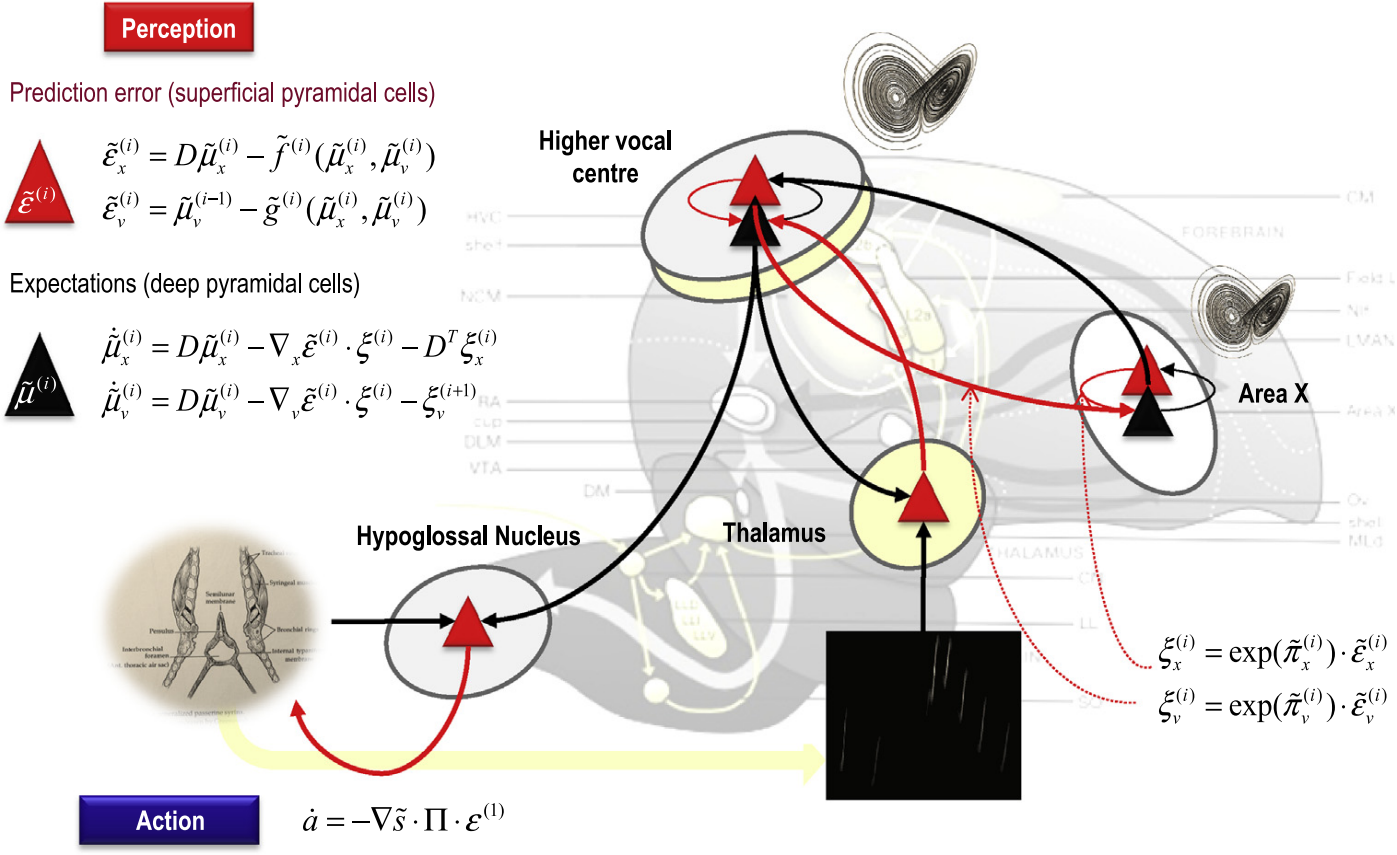
This figure summarizes hierarchical neuronal message passing in predictive coding using the (simplified) neuroanatomy of a songbird. Neuronal activity encodes expectations about the causes of sensory input, where these expectations minimize prediction error. Prediction error is the difference between (ascending) sensory input and (descending) predictions of that input. This minimization rests upon recurrent neuronal interactions among different levels of the cortical hierarchy. The available evidence suggests that superficial pyramidal cells (red triangles) compare the expectations (at each level) with top–down predictions from deep pyramidal cells (black triangles) of higher levels. **Left panel**: these equations represent the neuronal dynamics implicit in predictive coding. Prediction errors at the *i*th level of the hierarchy are simply the difference between the expectations encoded at that level and top–down predictions of those expectations. The expectations *per se* are driven by prediction errors so that they perform a gradient ascent on the sum of squared (precision weighted) prediction error. See the appendix for a detailed explanation of these (simplified) equations. **Right panel**: this provides a schematic example in the auditory system of a songbird: it shows the putative cells of origin of ascending or forward connections that convey (precision weighted) prediction errors (red arrows) and descending or backward connections (black arrows) that construct predictions. In this example, area X sends predictions to the higher vocal centre, which projects to the auditory thalamus. However, the higher vocal centre also sends proprioceptive predictions to the hypoglossal nucleus, which are passed to the syrinx to generate vocalisation through classical reflexes. These predictions can be regarded as motor commands, while the descending predictions of auditory input correspond to corollary discharge. Note that every top–down prediction is reciprocated with a bottom–up prediction error to ensure predictions are constrained by sensory information. Please see the Appendix for a description of the equations and a definition of the variables. (For interpretation of the references to colour in this figure legend, the reader is referred to the web version of this article.)

In summary, predictive coding represents a biologically plausible scheme for updating beliefs about states of the world using sensory samples: see Fig. 1. In this setting, cortical hierarchies are a neuroanatomical embodiment of how sensory signals are generated; for example, a face generates luminance surfaces that generate textures and edges and so on, down to retinal input. This form of hierarchical inference explains a large number of anatomical and physiological facts as reviewed elsewhere (Adams et al., 2012; Bastos et al., 2012; Friston, 2008). In brief, it explains the hierarchical nature of cortical connections; the prevalence of backward connections and explains many of the functional and structural asymmetries in the extrinsic (between region) connections that link hierarchical levels (Zeki & Shipp, 1988). These asymmetries include the laminar specificity of forward and backward connections, the prevalence of nonlinear or modulatory backward connections (that embody interactions and nonlinearities inherent in the generation of sensory signals) and their spectral characteristics – with fast (e.g., gamma) activity predominating in forward connections and slower (e.g., beta) frequencies that accumulate evidence (prediction errors) ascending from lower levels.

### 2.2 Precision engineered message passing

One can regard ascending prediction errors as broadcasting ‘newsworthy’ information that has yet to be explained by descending predictions. However, the brain has to select the channels it listens to – by adjusting the volume or *gain* of prediction errors that compete to update expectations in higher levels. Computationally, this gain corresponds to the precision or confidence associated with ascending prediction errors. However, to select prediction errors, the brain has to estimate and encode their precision (i.e., inverse variance). Having done this, prediction errors can then be weighted by their precision so that only precise information is accumulated and assimilated at high or deep hierarchical levels. As for all expectations, expected precision maximises Bayesian model evidence (see Appendix).

The broadcasting of precision-weighted prediction errors rests on gain control at a synaptic level (Moran et al., 2013). This neuromodulatory gain control corresponds to a (Bayes-optimal) encoding of precision in terms of the excitability of neuronal populations reporting prediction errors (Feldman & Friston, 2010; Shipp et al., 2013). This may explain why superficial pyramidal cells have so many synaptic gain control mechanisms; such as NMDA receptors and classical neuromodulatory receptors like D1 dopamine receptors (Braver, Barch, & Cohen, 1999; Doya, 2008; Goldman-Rakic, Lidow, Smiley, & Williams, 1992; Lidow, Goldman-Rakic, Gallager, & Rakic, 1991). Furthermore, it places excitation-inhibition balance in a prime position to mediate precision engineered message passing within and among hierarchical levels (Humphries, Wood, & Gurney, 2009). The dynamic and context sensitive control of precision has been associated with attentional gain control in sensory processing (Feldman & Friston, 2010; Jiang, Summerfield, & Egner, 2013) and has been discussed in terms of affordance in active inference and action selection (Cisek, 2007; Frank, Scheres, & Sherman, 2007; Friston et al., 2012). Crucially, the delicate balance of precision at different hierarchical levels has a profound effect on veridical inference – and may also offer a formal understanding of false inference in psychopathology (Adams, Stephan, Brown, Frith, & Friston, 2013; Fletcher & Frith, 2009; Friston, 2013). In what follows, we will see it has a crucial role in sensory attenuation.

### 2.3 Active inference

So far, we have only considered the role of predictive coding in perception through minimising surprise or prediction errors. However, there is another way to minimise prediction errors; namely, by re-sampling sensory inputs so that they conform to predictions; in other words, changing sensory inputs by changing the world through action. This is known as active inference (Friston et al., 2011). In active inference, action is regarded as the fulfilment of descending proprioceptive predictions by classical reflex arcs. In more detail, the brain generates continuous proprioceptive predictions about the expected location of the limbs and eyes – that are hierarchically consistent with the inferred state of the world. In other words, we believe that we will execute a goal-directed movement and this belief is unpacked hierarchically to provide proprioceptive and exteroceptive predictions entailed by our generative or forward model. These predictions are then fulfilled automatically by minimizing proprioceptive prediction errors at the level of the spinal cord and cranial nerve nuclei: see (Adams et al., 2012) and Fig. 1. Mechanistically, descending proprioceptive predictions provide a target or *set point* for peripheral reflex arcs – that respond by minimising (proprioceptive) prediction errors.

The argument here is that the same inferential mechanisms underlie apparently diverse functions (action, cognition and perception) but are essentially the same; for example, in the visual cortex for vision, the insula for interoception, the motor cortex for movement and proprioception. Crucially, because these modality-specific systems are organised hierarchically, they are all contextualised by the same conceptual (amodal) predictions. In other words, action and perception are facets of the same underlying imperative; namely to minimize hierarchical prediction errors through selective sampling of sensory inputs. However, there is a potential problem here:

### 2.4 Action and sensory attenuation

If proprioceptive prediction errors can be resolved by classical reflexes or changing (proprioceptive) expectations, how does the brain adjudicate between these two options? The answer may lie in the precision afforded to proprioceptive prediction errors and the consequences of movement sensed in other modalities. In order to engage classical reflexes, it is necessary to increase their gain through augmenting the precision of (efferent) proprioceptive prediction errors that drive neuromuscular junctions. However, to preclude (a veridical) inference that the movement has not yet occurred, it is necessary to attenuate the precision of (afferent) prediction errors that would otherwise update expectations or beliefs about the motor plant (Friston et al., 2011). Put simply, my prior belief that I am moving can be subverted by sensory evidence to the contrary; thereby precluding movement. In short, it is necessary to attenuate all the sensory consequences of moving – leading to an active inference formulation of sensory attenuation – the psychological phenomena that the magnitudes of self-made sensations are perceived as less intense (Chapman, Bushnell, Duncan, & Lund, 1987; Cullen, 2004).

In Fig. 1, we have omitted the (afferent) proprioceptive prediction error from the hypoglossal nucleus: see (Shipp et al., 2013) for discussion of this omission and the agranular nature of motor cortex. This renders descending proprioceptive predictions motor commands, where the accompanying exteroceptive predictions become corollary discharge. The ensuing motor control is effectively *open loop*. However, the hierarchical generation of proprioceptive predictions is contextualised by sensory input in other modalities – that register the sensory consequences of movement. It is these sensory consequences that are transiently attenuated during movement. In summary, to act, one needs to temporarily suspend attention to the consequences of action, in order to articulate descending predictions (Brown, Adams, Parees, Edwards, & Friston, 2013). Later, we will see that sensory attenuation plays a key role in communication.

### 2.5 Birdsong and attractors

This section introduces the simulations of birdsong that we will use to illustrate active inference and communication in subsequent sections. We are not interested in modelling birdsong *per se* – or the specifics of birdsong communication. Birdsong is used here as a minimal (metaphorical) example of biologically plausible communication with relatively rich dynamics: noting that there is an enormous literature on the neurobiology and physics of birdsong (Mindlin & Laje, 2005), some of which is particularly pertinent to active inference; e.g., (Hanuschkin, Ganguli, & Hahnloser, 2013). We have used birdsong in previous work to illustrate perceptual categorisation and other phenomena. Here, we focus on omission-related responses to illustrate the basic nature of predictive coding of hierarchically structured sensory dynamics. The basic idea here is that the environment unfolds as an ordered sequence of states, whose equations of motion induce attractor manifolds that contain sensory trajectories. If we consider the brain has a generative model of these trajectories, then we would expect to see attractors in neuronal dynamics that are trying to predict sensory input. This form of generative model has a number of plausible characteristics:

Models based upon attractors can generate and therefore encode structured sequences of events, as states flow over different parts of the manifold. These sequences can be simple, such as the quasi-periodic attractors of central pattern generators or can exhibit complicated sequences of the sort associated with itinerant dynamics (Breakspear & Stam, 2005; Rabinovich, Huerta, & Laurent, 2008). Furthermore, hierarchically deployed attractors enable the brain to predict or represent different categories of sequences. This is because any low-level attractor embodies a family of trajectories. A natural example here would be language (Jackendoff, 2002). This means it is possible to generate and represent sequences of sequences and, by induction sequences of sequences of sequences etc. (Kiebel, von Kriegstein, Daunizeau, & Friston, 2009). In the example below, we will try to show how attractor dynamics furnish generative models of sensory input, which behave much like real brains, when measured electrophysiologically: see (Friston & Kiebel, 2009) for implementational details.

### 2.6 A synthetic songbird

**Fig. 2:**
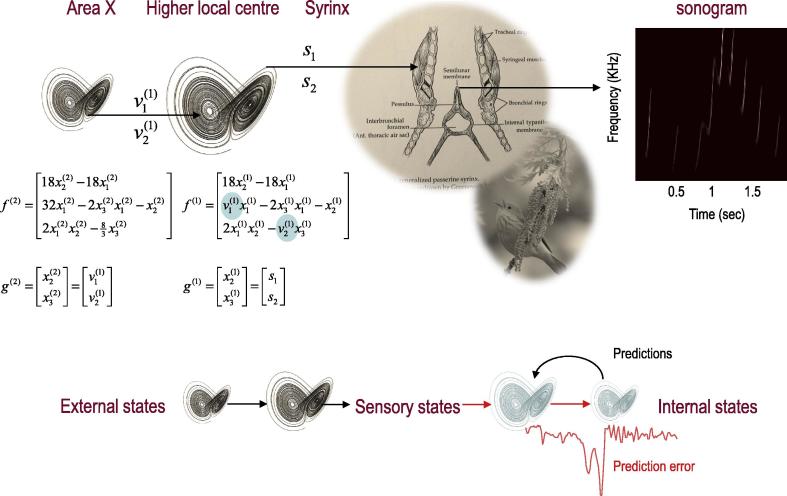
Schematic showing the construction of the generative model for birdsongs. The upper panel illustrates the generative model that comprises two Lorenz attractors, where the higher attractor delivers two control parameters (cyan circles) to a lower level attractor, which, in turn, delivers two control parameters to a synthetic syrinx to produce amplitude and frequency modulated stimuli. This stimulus is represented as a sonogram in the right panel. The equations represent the hierarchical dynamic model in the form described in the appendix. The lower panel illustrates the setup that we will be using, where sensory signals are generated using the scheme in the upper panel and are subsequently used to produce predictions and prediction errors (of the sort that can be measured empirically).

The example used here deals with the generation and recognition of birdsongs. We imagine that birdsongs are produced by two time-varying control parameters that control the frequency and amplitude of vibrations of the syrinx of a songbird (see Fig. 2). There has been an extensive modelling effort using attractor models at the biomechanical level to understand the generation of birdsong (Mindlin & Laje, 2005). Here we use attractors at a higher level to provide time-varying control over the resulting sonograms. We drive the syrinx with two states of a Lorenz attractor, one controlling the frequency (between two to five kHz) and the other controlling the amplitude or volume. The parameters of the Lorenz attractor were chosen to generate a short sequence of chirps every second or so. To give the generative model a hierarchical structure, we placed a second Lorenz attractor, whose dynamics were an order of magnitude slower, over the first (seconds as opposed to 100 ms or so); such that the states of the slower attractor change the manifold of the fast attractor. This manifold could range from a fixed-point attractor, where the states collapse to zero; through to quasi-periodic and chaotic behaviour. Because higher states evolve more slowly, they switch the lower attractor on and off, generating songs, where each song comprises a series of distinct chirps (see Fig. 2).

### 2.7 Omission and violation of predictions

To illustrate the predictive nature of predictive coding, song recognition was simulated by integrating the predictive coding scheme above (equations in Fig. 1). These simulations used a standard integration scheme (**spm_ADEM.m**) described in detail in the appendix. The simulations reported in this paper can be reproduced by downloading SPM (http://www.fil.ion.ucl.ac.uk/spm/) and typing DEM to access the graphical user interface for the DEM Toolbox (**Birdsong duet**).

**Fig. 3:**
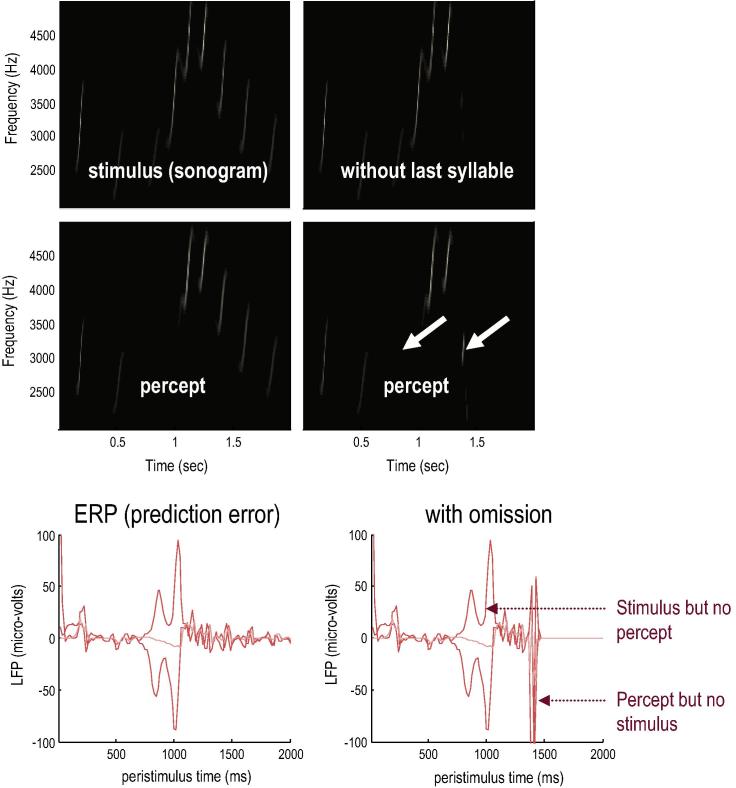
Omission-related responses: The left panels show the original song and responses evoked. The right panels show the equivalent dynamics on omission of the last chirps. The top panels show the stimulus and the middle panels the corresponding percept in sonogram format. The interesting thing to note here is the occurrence of an anomalous percept after termination of the song on the lower right. This corresponds roughly to the chirp that would have been perceived in the absence of omission. The lower panels show the corresponding (precision weighted) prediction error under the two stimuli at both levels. A comparison of the two reveals a burst of prediction error when a stimulus is missed and at the point that the stimulus terminates – even when there is no stimulus present at this time. The darker lines correspond to prediction error at the first level and the lighter lines correspond to prediction error at the second level.

A sonogram was produced using the above composition of Lorentz attractors (Fig. 2) and played to a synthetic bird – who tried to infer the underlying hidden states of the first and second level attractors. These attractors are associated with the higher local centre and area X in Fig. 1. Crucially, we presented two songs to the bird, with and without the final chirps. The corresponding sonograms and percepts (predictions) are shown with their prediction errors in Fig. 3.

The left panels show the stimulus and percept, while the right panels show the stimulus and responses to omission of the last chirps. These results illustrate two important phenomena. First, there is a vigorous expression of prediction error after the song terminates abruptly. This reflects the dynamical nature of the recognition process because, at this point, there is no sensory input to predict. In other words, the prediction error is generated entirely by the predictions afforded by the dynamic model of sensory input. It can be seen that this prediction error (with a percept but no stimulus) is larger than the prediction error associated with the third and fourth stimuli that are not perceived (stimulus but no percept). Second, there is a transient percept when the omitted chirp should have occurred. Its frequency is too low but its timing is preserved in relation to the expected stimulus train. This is an interesting stimulation from the point of view of ERP studies of omission-related responses; particularly given that non-invasive electromagnetic signals arise largely from superficial pyramidal cells – which are the cells thought to encode prediction error (Bastos et al., 2012). Empirical studies of this characteristic response to violations provide clear evidence for the predictive capacity of the brain (Bendixen, SanMiguel, & Schröger, 2012).

### 2.8 Creating your own sensations – a synthetic soliloquy

We now consider the production of birdsong using the same model used for perception above. In the preceding simulations, descending predictions of exteroceptive (auditory) sensations were used to construct prediction errors that enabled perceptual inference and recognition of deep (hierarchical) structure in the sensory stream. However, in Fig. 1, there are also descending proprioceptive predictions to the hypoglossal region that elicit action through classical reflex arcs. This means the synthetic bird could, in principle, sing to itself – predicting both the exteroceptive and proprioceptive consequences of its action. This was precluded in the above simulations by setting the precision or gain of efferent proprioceptive prediction errors (that drive motor reflexes) to a very low value (a log precision of minus eight), in contrast to auditory prediction errors (with a log precision of two). This means that the precision weighted prediction errors do not elicit any action or birdsong, enabling the bird to listen to its companion. So what would happen if we increased the precision of proprioceptive prediction errors?

**Fig. 4:**
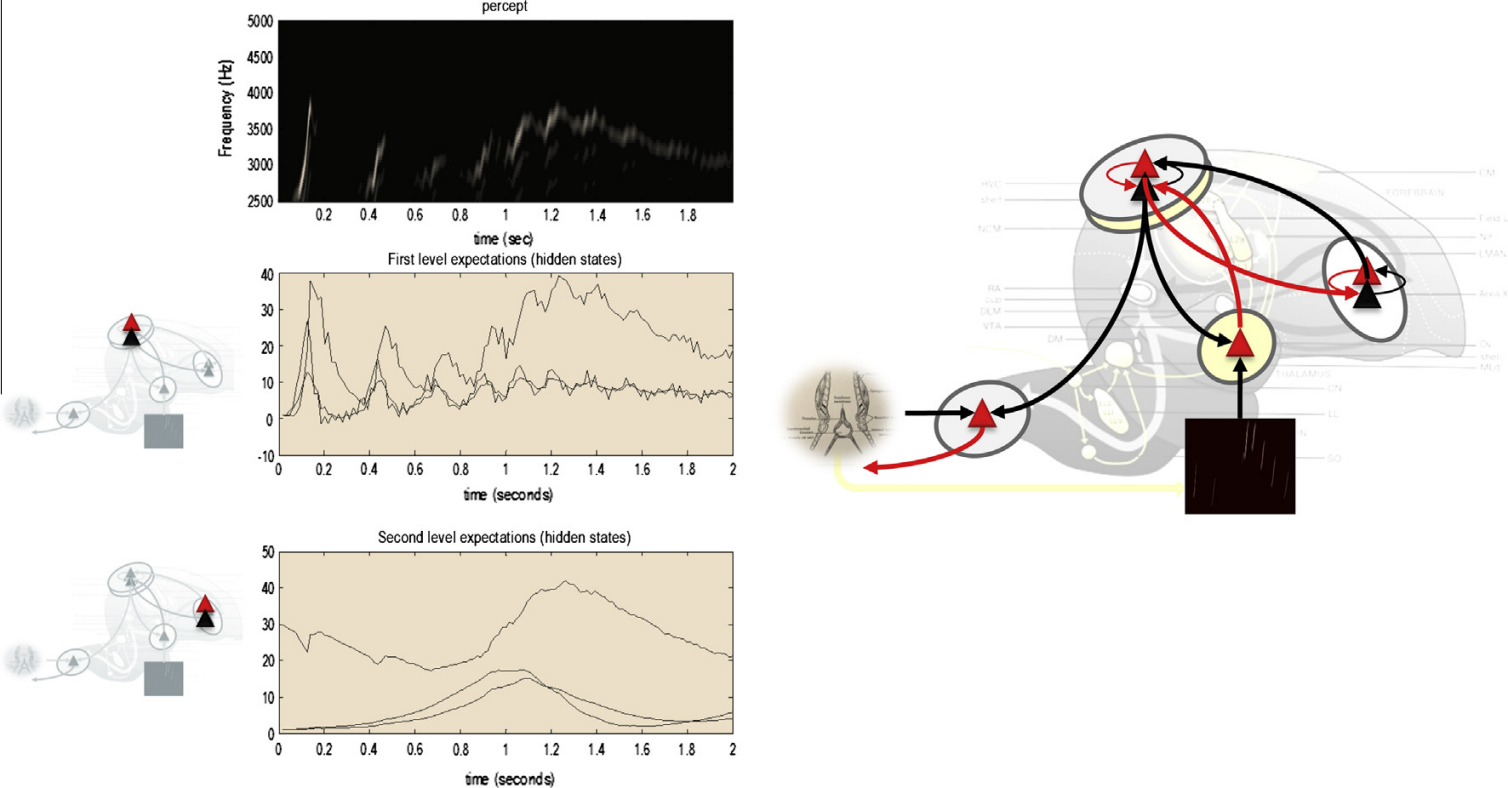
creating one’s own sensations. This figure shows the results of a simulation in which the precision (gain) of proprioceptive prediction errors was increased from a log precision of −8 to 8 (highlighted by the insert on the right). This effectively switches on reflex arcs that respond to descending proprioceptive predictions from the higher vocal centre, causing the previously perceived song to be articulated. However, when we run the simulations (ensuring that the bird can hear itself) the ensuing song is unrecognisable. This is shown in the upper panel in terms of a bizarre looking sonogram that has lost the deep hierarchical structure and tempo of the sonograms in the previous figures. The middle (resp. lower) panel shows posterior expectations about hidden states in the first (resp. second) hierarchical levels. The dynamics characteristic of the Lorentz attractor have been destroyed and low amplitude high-frequency fluctuations are evident in these traces. These reflect the impact of precise prediction errors due to sensorimotor delays in the enactment of predictions.

One might anticipate that descending (multimodal) predictions from the higher vocal centre would cause the bird to sing – and predict the consequences of its own action – thereby eliciting a soliloquy. In fact, when the proprioceptive precision is increased (to a log precision of eight) something rather peculiar happens: Fig. 4 shows that a rather bizarre sonogram is produced, with low amplitude, high-frequency components and a loss of the song’s characteristic structure. The explanation for this failure lies in the sensorimotor delays inherent in realising proprioceptive predictions. In other words, descending auditory predictions fail to account for the slight delay in self-made sensations. This results in perpetual (and precise) exteroceptive prediction errors that confound perceptual synthesis and associated action (singing). This resonates with the well-known disruptive effect of delayed auditory feedback on speech (Yates, 1963). Put simply, action and perception chase each other’s tails, never resolving the discrepancy between the actual and predicted consequences of action. Although it would be possible to include sensorimotor delays in the generative model – see (Perrinet & Friston, 2014) for an example oculomotor control – a simpler solution rests on sensory attenuation:

### 2.9 Sensory attenuation and action

**Fig. 5:**
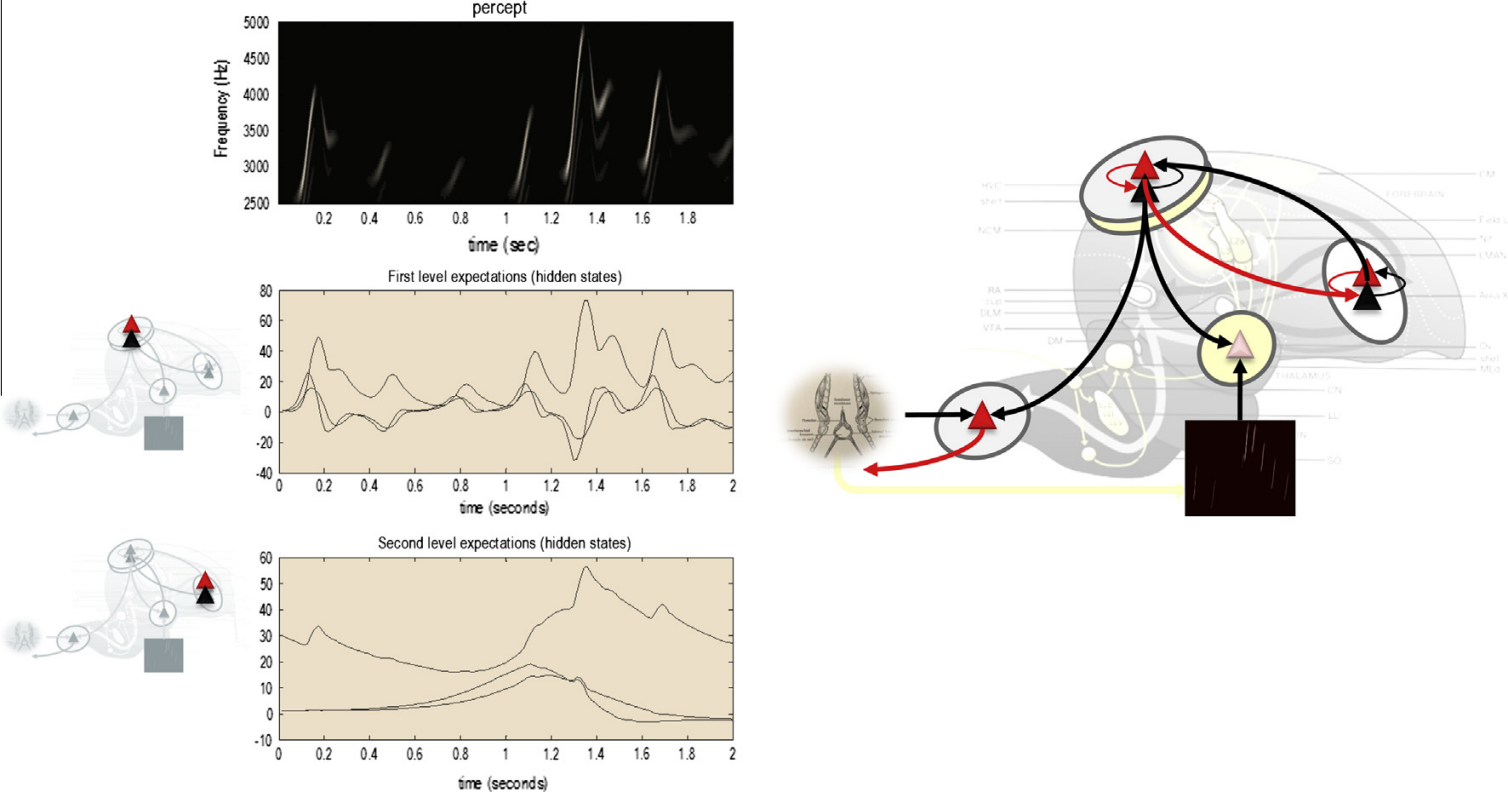
sensory attenuation during self-made acts. This figure uses the same format as Fig. 4 – and reports the same simulation. However, here, we have reduced the log precision of sensory (auditory) prediction errors from 2 to 0. This effectively prevents ascending – and irreducible – prediction errors due to sensorimotor delays from confounding the dynamics in the higher vocal centre. This means the higher vocal centre can provide veridical top–down proprioceptive predictions and elicit and uncorrupted birdsong. This example illustrates the permissive nature of sensory attenuation during the enactment of descending predictions through open loop control.

If auditory predictions are imprecise, by virtue of sensorimotor delays, then their precision should be attenuated. This is an example of sensory attenuation or attenuation of sensory precision (Brown et al., 2013). If we attenuate the auditory precision by a factor of exp(2) – by reducing the log precision from 2 to 0 – the bird is now able to produce a well-formed soliloquy that would be recognised by another bird: see Fig. 5. The attenuation of sensory precision corresponds effectively to attending away from the consequences of action. A nice example of this is our inability to perceive (attend to) optical flow produced by saccadic eye movements: when visual motion or flow is produced exogenously – say by gently palpating the eyeball – they can be perceived but not when produced by oculomotor action. This simple but remarkable fact was first noted by Bell in 1823 and subsequently Helmholtz in 1866 (Wade, 1978). Put simply, we cannot speak and listen at the same time (Numminen, Salmelin, & Hari, 1999). From the perspective of the oculomotor system, this suggests that saccadic eye movements are imperceptible palpations of the world that are only attended to once complete; c.f., active vision (Wurtz, McAlonan, Cavanaugh, & Berman, 2011). Heuristically, this suggests that active inference presents in one of two modes; either attending to sensations or acting during periods of sensory attenuation. It also suggests that behaviour such as speech (that rests upon preordained sequences) may only be articulated with open loop control – a loop that is opened by sensory attenuation.

### 2.10 Summary

These (and many other) simulations suggest that action and perception depend upon a delicate balance between the precision of proprioceptive and exteroceptive prediction errors that orchestrate the perceptual synthesis of sensations (caused by others) or realising sensory predictions (caused by self). It further suggests that certain behaviours (such as communication) are mediated by (transient) open loop control, which depends on sensory attenuation. This seems a plausible perspective on speech, in which violations or failures are generally recognised *post hoc* (e.g., slip of the tongue phenomena). It is interesting to speculate on the psychopathology that might attend a failure of sensory attenuation. In the first simulation above, we illustrated the failure to realise intended utterances when the precision of both proprioceptive and exteroceptive prediction errors were high. This might provide an interesting metaphor for failures of articulation; e.g., stuttering (Kalinowski, Armson, Rolandmieszkowski, Stuart, & Gracco, 1993). The converse pathology would be when both proprioceptive and exteroceptive prediction errors had too little precision, leading to psychomotor poverty and bradykinesia (e.g., Parkinson’s disease). See (Adams et al., 2013) for a more general discussion of precision in the context of psychopathology.

One might ask whether sensory attenuation means that one cannot hear oneself. In quantitative terms, simulations like those above suggest the attenuation of sensory position is only quantitative: in other words, if the precision of prediction errors at extrasensory levels is greater than sensory precision, then descending proprioceptive predictions can be enacted with impunity – despite irreducible sensory prediction errors. This means that one can register and subsequently attend to violations in the consequences of action. Furthermore, it is possible to simulate talking to oneself by noting that higher levels of the cortical hierarchy (e.g., area X) are receiving prediction errors from lower areas (e.g., the higher vocal centre). In this sense, higher areas listen to lower areas, which embody sensorimotor content that may, or may not be, articulated (depending upon whether proprioceptive gain or precision is high or low). In the next section, we exploit the notion of sensory attenuation and model two birds that listen and sing to each other.

## 3 A Duet for one

**Fig. 6:**
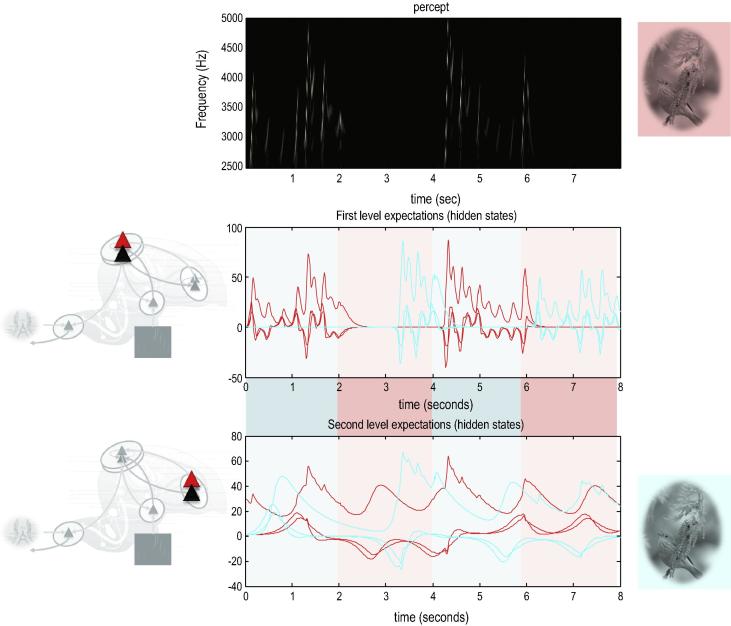
a soliloquy for two. In this simulation, two birds with the same generative models – but different initial expectations – sing for two seconds and then listen for any response. However, here, the birds cannot hear each other (e.g., they are too far apart) and the successive epochs of songs diverge due to the sensitivity to initial conditions implicit in these (chaotic) generative models. The upper panel shows the sonogram heard by the first (red) bird. Because this bird can only hear itself, the sonogram reflects the proprioceptive predictions based upon posterior expectations in the higher vocal centre (middle panel) and area X (lower panel). The posterior expectations for the first bird are shown in red as a function of time – and the equivalent expectations for the second bird are shown in blue. Note that when both birds are listening, their expectations at the first level fall to zero – because they do not hear anything and auditory input is attended to (i.e., has a relatively high precision). This does not destroy the slower dynamics in area X, which is able to generate the song again after the end of each listening period. Note also that the second (blue) bird takes a few hundred milliseconds before it starts singing. This is because it takes a little time for the posterior expectations to find the attractor manifold prescribed by the higher level control parameters. (For interpretation of the references to colour in this figure legend, the reader is referred to the web version of this article.)

**Fig. 7:**
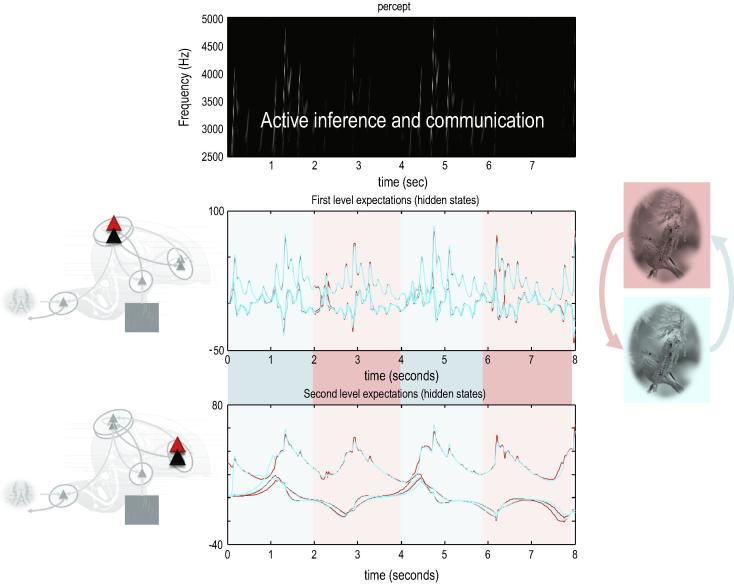
communication and generalised synchrony. This figure uses the same format as Fig. 6; however, here, we have juxtaposed the two birds so that they can hear each other. In this instance, the posterior expectations show identical synchrony at both the first and second hierarchical levels – as shown in the middle and lower panels respectively. Note that the sonogram is continuous over successive one second epochs – being generated alternately by the first and second bird.

Finally, we turn to the perceptual coupling or communication by simulating two birds that can hear themselves (and each other). Each bird listened for two seconds (with a low proprioceptive precision and a high exteroceptive precision) and then sang for two seconds (with high proprioceptive precision and attenuated auditory precision). Crucially, when one bird was singing the other was listening. We started the simulations with random initial conditions. This meant that if the birds cannot hear each other, the chaotic dynamics implicit in their generative models causes their expectations to follow independent trajectories, as shown in Fig. 6. However, if we move the birds within earshot – so that they can hear each other – they synchronise almost immediately. See Fig. 7. This is because the listening bird is quickly entrained by the singing bird to correctly infer the hidden (dynamical) states generating sensations. At the end of the first period of listening, the posterior expectations of both parents display identical synchrony, which enables the listening bird to take up the song, following on from where the other bird left off. This process has many of the hallmarks of *interactive alignment* in the context of joint action and dialogue (Garrod & Pickering, 2009).

Note that the successive epochs of song are not identical. In other words, the birds are not simply repeating what they have heard – they are pursuing a narrative embodied by the dynamical attractors (central pattern generators) in their generative models that have been synchronised through sensory exchange. As noted above, this means that both birds can sing from the same hymn sheet, preserving sequential and hierarchical structure in their shared narrative. It is this phenomenon – due simply to generalised (in this case identical) synchronisation of inner states – we associate with communication. The reason that synchronisation is identical is that both birds share the same prior expectations. In a companion paper we will illustrate how they *learn* each other’s attractors to promote identical synchronisation.

### 3.1 Summary

In summary, these illustrations show that generalised synchrony is an emergent property of coupling active inference systems that are trying to predict each other. In this context, it is interesting to consider what is being predicted. The sensations in Fig. 7 are continuous and (for both birds) are simply the consequences of some (hierarchically composed and dynamic) hidden states. But what do these states represent? One might argue that they correspond to a construct that drives the behaviour of one or other bird to produce the sensory consequences that are sampled. But which bird? The sensory consequences are generated, in this setting, by both birds. It therefore seems plausible to assign these hidden states to both birds and treat the agency as a contextual factor (that depends on sensory attenuation). In other words, from the point of view of one bird, the hidden states are amodal, generating proprioceptive and exteroceptive consequences that are inferred in exactly the same way over time; irrespective of whether sensory consequences are generated by itself or another. The agency or source of sensory consequences is determined not by the hidden states *per se* – but by fluctuations in sensory attenuation (and proprioceptive precision). In this sense, the expectations are without agency. This agent-less aspect may be a quintessential aspect of shared perspectives and communication.

## 4 Conclusion

The arguments in this paper here offer a somewhat unusual solution to the theory of mind problem. This solution replaces the problem of inferring another’s mental state with inferring what state of mind one would be into produce the same sensory consequences: c.f., ideomotor theory (Pfister, Melcher, Kiesel, Dechent, & Gruber, 2014). Conceptually, this is closely related to Bayesian accounts of the mirror neuron system – in which generative models are used to both produce action and infer the intentions of actions observed in others (Kilner et al., 2007). The basic idea is that internal or generative models used to infer one’s own behaviour can be deployed to infer the beliefs (e.g., intentions) of another – provided both parties have sufficiently similar generative models. The example of communication considered above goes slightly further than this – and suggests that prior beliefs about the causes of behaviour (and its consequences) are not necessarily tied to any particular agent: rather, they are used to recognise canonical behaviours that are intermittently generated by oneself and another. In other words, the only reason that the simulations worked was because both birds were equipped with the same generative model. This perspective renders representations of intentional set and narratives almost Jungian in nature – presupposing a collective narrative that is shared among communicating agents (including oneself). For example, when in conversation or singing a duet, our beliefs about the (proprioceptive and auditory) sensations we experience are based upon expectations about the song. These beliefs transcend agency in the sense that the song (e.g., hymn) does not belong to you or me – agency just contextualises its expression.

In this paper, we have focused on establishing the basic phenomenology of generalised synchrony in the setting of active inference. Interpreting this inference in terms of theory of mind presupposes that agents share a similar generative model of communicative behaviour. In a companion paper (Friston & Frith, 2015), we demonstrate how communication facilitates long-term changes in generative models that are trying to predict each other. In other words, communication induces perceptual learning, ensuring that both agents come to share the same model. This is almost a self evident consequence of learning or acquiring a model: if the objective of learning is to minimise surprise or maximise the predictability of sensations, then this is assured if both agents converge on the same model to predict each other.

Clearly, we are not saying that theory of mind involves some oceanic state in which ego boundaries are dissolved. This is because using generative models to predict one’s own behaviour and the behaviour of others requires a careful orchestration of sensory precision and proprioceptive gain that contextualises the inference. This means, the generative model must comprise beliefs about the deployment of precision; including when to listen and when to talk; c.f., *turn* taking (Wilson & Wilson, 2005). We have not dealt with this aspect of communication here. However, it is clearly an important issue that probably rests on prosocial learning. It is interesting to note that a failure of sensory attenuation – in particular the relative strength of sensory and prior (extrasensory) precision – has been proposed as the basis of autism – whose cardinal features include an impoverished theory of mind (Happe & Frith, 2006; Lawson, Rees, & Friston, 2014; Pellicano & Burr, 2012; Van de Cruys et al., 2014). Furthermore, we have only considered a very elemental form of communication (and implicit theory of mind). We have not considered the reflective processing that may accompany its deliberative (conscious) aspects. This is an intriguing and challenging area (particularly from the point of view of modelling) that calls on things like deceit: e.g., (Talwar & Lee, 2008). Finally, we have not addressed non-verbal communication in motor and autonomic domains. A particularly interesting issue here is interoceptive inference (Seth, 2013), and how visual cues about the emotional states of others can be interpreted in relation to generative models of our own interoceptive cues: see (Kanaya, Matsushima, & Yokosawa, 2012).

So far, in our discussion of communication, we have emphasised the importance of turn taking – and how this can lead to alignment of posterior expectations and *identical* synchronisation of inner states. However, one of the most important functions of communication is to enable one person to change another. For example, we can change another’s behaviour by giving them instructions in an experiment, or we can change their minds by helping them to understand a new concept (like free energy). For this aspect of communication there is an obvious asymmetry in the interaction, since the aim is to transfer information from one mind to another. Nevertheless, the basic features of the process remain as described above – and the asymmetric exchange will call upon *generalised* synchronisation. In this context, the naive speaker may assign greater salience or precision to predictions errors to update his (imprecise) priors about the nature of the new concept, while the knowledgeable agent will try to change the state of his listener. For the knowledgeable speaker, prediction errors indicate that his listener has still not understood (e.g., what must he be thinking to say that). For the ignorant speaker, prediction errors indicate that his concept is still not quite right: see Fig. 9 and (Frith & Wentzer, 2013) for discussion of the implicit hermeneutics. But the end result of the interaction will be a generalised synchronisation between the speakers. The emergence of such synchronisation indicates that the concept has been successfully communicated – and both parties can accurately predict what the other will say. As a result Chris’s concept (of free energy) will have changed a lot, but Karl’s will have changed a little as a result of communicating with Chris.

From a mathematical perspective, we have promoted generalised synchrony (or synchronisation of chaos) as a mathematical image of communication. Furthermore, we have suggested that this synchronisation is an inevitable and emergent property of coupling two systems that are trying to predict each other. This assertion rests on the back story to active inference; namely, the free energy principle (Friston et al., 2006). Variational free energy provides an upper bound on surprise (i.e., free energy is always greater than surprise). This means that minimising free energy through active inference implicitly minimises surprise (or prediction errors), which is the same as maximising Bayesian model evidence. It is fairly easy to show that any measure-preserving dynamical system – including ourselves – that possesses a Markov blanket (here sensations and action) will appear to minimise surprise (Friston, 2013). The long-term average of surprise is called entropy, which means minimising surprise minimises (information theoretic) entropy. This is important because it means action (and perception) will appear to minimise the entropy of sensory samples, which are caused by external states. In turn, this means internal states will inevitably exhibit a generalised synchronisation with a system’s internal states. All that we have done in this paper is to associate the external states with the internal states of another agent. So why is generalised synchrony inevitable?

**Fig. 8:**
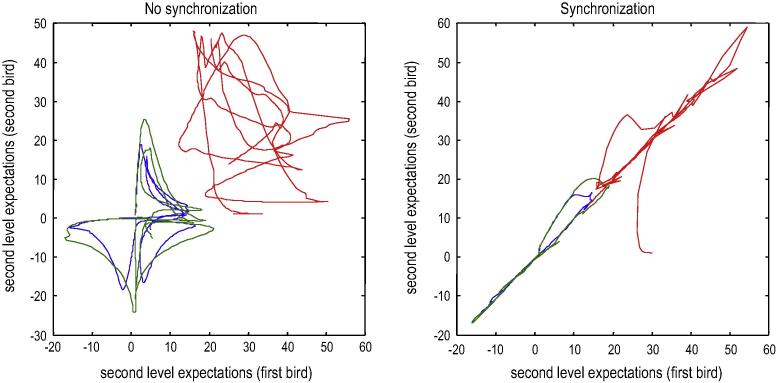
the synchronisation manifold. These graphs plot the second level (area X) expectations in the second bird against the equivalent expectations in the first. The left panel shows chaotic and uncoupled dynamics when the birds cannot hear each other, while the right panel shows the generalised (identical) synchrony that emerges when the birds exchange sensory signals. The different colours correspond to the three hidden states for each bird. The synchronisation manifold for identical synchronisation would correspond to the X equals Y line.

**Fig. 9:**
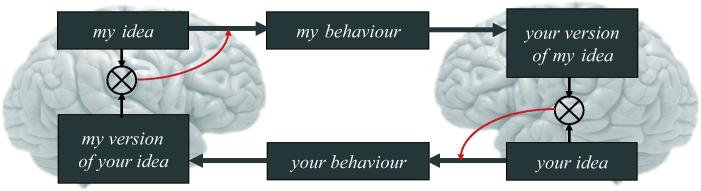
A predictive coding formulation of the Hermeneutic Circle.

This follows from the measure-preserving nature of coupled dynamical systems, which implies that internal states, external states – and the Markov blanket that separates them – possesses something called a random dynamical attractor. This attracting set of states plays the role of a *synchronisation manifold* that gives rise to generalise synchrony. The synchronisation manifold is just a set of states to which states are attracted to and thereafter occupy. All other points in the joint state space of internal and external states are unstable and will eventually end up on the synchronisation manifold. The simplest example of a synchronisation manifold would be the X equals Y line on a graph plotting an external state against an internal state (see Fig. 8). This corresponds to identical synchronisation. In other words, external and internal states track each other or – in the current context – both agents become identically synchronised. Crucially, the attractor (which contains the synchronisation manifold) has a low measure or volume. This is usually characterised in terms of the (fractional) dimensionality of the attractor. For example, in Fig. 8 the synchronisation manifold collapses to one dimension with the emergence of generalised synchrony. A measure of the attractor’s volume is provided by its *measure theoretic* entropy (Sinai, 1959). Although formally distinct from *information theoretic* entropy, both reflect the volume of the attracting set of dynamical states occupied by the systems in question. This means that minimising free energy (information theoretic entropy) reduces the volume (measure theoretic entropy) of the random dynamical attractor (synchronisation manifold); thereby inducing generalise synchrony.

In conclusion, if the universe comprised me and you – and we were measure-preserving – then your states and my states have to be restricted to an attracting set of states that is small relative to all possible states we could be in. This attracting set enforces a generalised synchrony in the sense that the state you are in imposes constraints on states I occupy. It is in this sense that generalised synchrony is a fundamental aspect of coupled dynamical systems that are measure (volume) preserving. Furthermore, if we are both trying to minimise the measure (volume) of our attracting set (by reducing surprise or entropy), then that synchronisation will be more manifest. The notion of generalised synchrony may lend a formal backdrop to observations of synchronisation of brain activity between agents during shared perspective taking (Moll & Meltzoff, 2011). For example, functional magnetic resonance imaging, suggests that internal action simulation synchronizes action–observation networks across individuals (Nummenmaa et al., 2014). The arguments above suggest that synchronisation is not only fundamental for a shared experience of the world – it is a fundamental property of the world that we constitute.

## 5 Conflict of interest statement

The authors declare no conflicts of interest.

## Appendix A Appendix

This brief description of generalized predictive coding is based on (Feldman & Friston, 2010). A more technical description can be found in (Friston, Stephan, Li, & Daunizeau, 2010). This scheme is based on three assumptions:

• The brain minimizes a free energy of sensory inputs defined by a generative model.
• The generative model used by the brain is hierarchical, nonlinear and dynamic.
• Neuronal firing rates encode the expected state of the world, under this model.

Free energy is a quantity from statistics that measures the quality of a model in terms of the probability that it could have generated observed outcomes. This means that minimizing free energy maximizes the Bayesian evidence for the generative model. The second assumption is motivated by noting that the world is both dynamic and nonlinear and that hierarchical causal structure emerges inevitably from a separation of spatial and temporal scales. The final assumption is the Laplace assumption that leads to the simplest and most flexible of all neural codes.

Given these assumptions, one can simulate a whole variety of neuronal processes by specifying the particular equations that constitute the brain’s generative model. In brief, these simulations use differential equations that minimize the free energy of sensory input using a generalized gradient descent.

(A.1) $$\overset{˙}{\overset{˜}{\mu }}(t)=D\overset{˜}{\mu }(t)-\partial_{\overset{˜}{\mu }}F(\overset{˜}{s}\text{,}\overset{˜}{\mu })$$

These differential equations say that neuronal activity encoding posterior expectations about (generalized) hidden states of the world $\overset{˜}{\mu }=(\mu \text{,}\mu^{\prime }\text{,}\mu^{\prime \prime }\text{,}\ldots )$ reduce free energy – where free energy $F(\overset{˜}{s}\text{,}\overset{˜}{\mu })$ is a function of sensory inputs $\overset{˜}{s}=(s\text{,}s^{\prime }\text{,}s^{\prime \prime }\text{,}\ldots )$ and neuronal activity. This is known as generalized predictive coding or Bayesian filtering. The first term is a prediction based upon a differential matrix operator *D* that returns the generalized motion of expected hidden states $D\overset{˜}{\mu }=(\mu \text{,}\mu^{\prime }\text{,}\mu^{\prime \prime }\text{,}\ldots )$. The second (correction) term is usually expressed as a mixture of prediction errors that ensures the changes in posterior expectations are Bayes-optimal predictions about hidden states of the world. To perform neuronal simulations under this scheme, it is only necessary to integrate or solve Eq. (A.1) to simulate the neuronal dynamics that encode posterior expectations. Posterior expectations depend upon the brain’s generative model of the world, which we assume has the following hierarchical form:

(A.2) $$\begin{array}{c} s=g^{\left(1\right)}(x^{\left(1\right)}\text{,}v^{\left(1\right)})+\exp\left(-\frac{1}{2}\pi_{v}^{\left(1\right)}(x^{\left(1\right)}\text{,}v^{\left(1\right)})\right)\cdot \omega_{v}^{\left(1\right)}\\ {\overset{˙}{x}}^{\left(1\right)}=f^{\left(1\right)}(x^{\left(1\right)}\text{,}v^{\left(1\right)})+\exp\left(-\frac{1}{2}\pi_{x}^{\left(1\right)}(x^{\left(1\right)}\text{,}v^{\left(1\right)})\right)\cdot \omega_{x}^{\left(1\right)}\\ \vdots \\ v^{\left(i-1\right)}=g^{\left(i\right)}(x^{\left(i\right)}\text{,}v^{\left(i\right)})+\exp\left(-\frac{1}{2}\pi_{v}^{\left(i\right)}(x^{\left(i\right)}\text{,}v^{\left(i\right)})\right)\cdot \omega_{v}^{\left(i\right)}\\ {\overset{˙}{x}}^{\left(i\right)}=f^{\left(i\right)}(x^{\left(i\right)}\text{,}v^{\left(i\right)})+\exp\left(-\frac{1}{2}\pi_{x}^{\left(i\right)}(x^{\left(i\right)}\text{,}v^{\left(i\right)})\right)\cdot \omega_{x}^{\left(i\right)}\\ \vdots \end{array}$$

This equation describes a probability density over the sensory and hidden states that generate sensory input. Here, the hidden states have been divided into hidden states and causes $\left(x^{\left(i\right)}\text{,}v^{\left(i\right)}\right)$ at the *i*th level within the hierarchical model. Hidden states and causes are abstract variables that the brain uses to explain or predict sensations – like the motion of an object in the field of view.

In these models, hidden causes link hierarchical levels, whereas hidden states link dynamics over time. Here, $\left(f^{\left(i\right)}\text{,}g^{\left(i\right)}\right)$ are nonlinear functions of hidden states and causes that generate hidden causes for the level below and – at the lowest level – sensory inputs. Random fluctuations in the motion of hidden states and causes $\left(\omega_{x}^{\left(i\right)}\text{,}\omega_{v}^{\left(i\right)}\right)$ enter each level of the hierarchy. Gaussian assumptions about these random fluctuations make the model probabilistic. They play the role of sensory noise at the first level and induce uncertainty at higher levels. The amplitudes of these random fluctuations are quantified by their precisions that may depend upon the hidden states or causes through their log-precisions $\left(\pi_{x}^{\left(i\right)}\text{,}\pi_{v}^{\left(i\right)}\right)$ .

Given the form of the generative model (Eq. (A.2)) we can now write down the differential equations (Eq. (A.1)) describing neuronal dynamics in terms of (precision-weighted) prediction errors. These errors represent the difference between posterior expectations and predicted values, under the generative model (using $A\cdot B≜\Delta =A^{T}B$ and omitting higher-order terms):

(A.3) $$\begin{array}{c} {\overset{˙}{\overset{˜}{\mu }}}_{x}^{\left(i\right)}=D{\overset{˜}{\mu }}_{x}^{\left(i\right)}+\left(\frac{\partial {\overset{˜}{g}}^{\left(i\right)}}{\partial {\overset{˜}{\mu }}_{x}^{\left(i\right)}}-\frac{1}{2}{\overset{˜}{\varepsilon }}_{v}^{\left(i\right)}\frac{\partial {\overset{˜}{\pi }}_{v}^{\left(i\right)}}{\partial {\overset{˜}{\mu }}_{x}^{\left(i\right)}}\right)\cdot \xi_{v}^{\left(i\right)}+\left(\frac{\partial {\overset{˜}{f}}^{\left(i\right)}}{\partial {\overset{˜}{\mu }}_{x}^{\left(i\right)}}-\frac{1}{2}{\overset{˜}{\varepsilon }}_{x}^{\left(i\right)}\frac{\partial {\overset{˜}{\pi }}_{x}^{\left(i\right)}}{\partial {\overset{˜}{\mu }}_{x}^{\left(i\right)}}\right)\cdot \xi_{x}^{\left(i\right)}+\frac{\partial \mathrm{tr}({\overset{˜}{\pi }}_{v}^{\left(i\right)}+{\overset{˜}{\pi }}_{x}^{\left(i\right)})}{\partial {\overset{˜}{\mu }}_{x}^{\left(i\right)}}-D^{T}\xi_{x}^{\left(i\right)}\\ {\overset{˙}{\overset{˜}{\mu }}}_{v}^{\left(i\right)}=D{\overset{˜}{\mu }}_{v}^{\left(i\right)}+\left(\frac{\partial {\overset{˜}{g}}^{\left(i\right)}}{\partial {\overset{˜}{\mu }}_{v}^{\left(i\right)}}-\frac{1}{2}{\overset{˜}{\varepsilon }}_{v}^{\left(i\right)}\frac{\partial {\overset{˜}{\pi }}_{v}^{\left(i\right)}}{\partial {\overset{˜}{\mu }}_{v}^{\left(i\right)}}\right)\cdot \xi_{v}^{\left(i\right)}+\left(\frac{\partial {\overset{˜}{f}}^{\left(i\right)}}{\partial {\overset{˜}{\mu }}_{x}^{\left(i\right)}}-\frac{1}{2}{\overset{˜}{\varepsilon }}_{x}^{\left(i\right)}\frac{\partial {\overset{˜}{\pi }}_{x}^{\left(i\right)}}{\partial {\overset{˜}{\mu }}_{v}^{\left(i\right)}}\right)\cdot \xi_{x}^{\left(i\right)}+\frac{\partial \mathrm{tr}({\overset{˜}{\pi }}_{v}^{\left(i\right)}+{\overset{˜}{\pi }}_{x}^{\left(i\right)})}{\partial {\overset{˜}{\mu }}_{v}^{\left(i\right)}}-\xi_{v}^{\left(i+1\right)}\\ \xi_{x}^{\left(i\right)}=\exp({\overset{˜}{\pi }}_{x}^{\left(i\right)})\cdot {\overset{˜}{\varepsilon }}_{x}^{\left(i\right)}\\ \xi_{v}^{\left(i\right)}=\exp({\overset{˜}{\pi }}_{v}^{\left(i\right)})\cdot {\overset{˜}{\varepsilon }}_{v}^{\left(i\right)}\\ {\overset{˜}{\varepsilon }}_{x}^{\left(i\right)}=D{\overset{˜}{\mu }}_{x}^{\left(i\right)}-{\overset{˜}{f}}^{\left(i\right)}({\overset{˜}{\mu }}_{x}^{\left(i\right)}\text{,}{\overset{˜}{\mu }}_{v}^{\left(i\right)})\\ {\overset{˜}{\varepsilon }}_{v}^{\left(i\right)}={\overset{˜}{\mu }}_{v}^{\left(i-1\right)}-{\overset{˜}{g}}^{\left(i\right)}({\overset{˜}{\mu }}_{x}^{\left(i\right)}\text{,}{\overset{˜}{\mu }}_{v}^{\left(i\right)}) \end{array}$$

This produces a relatively simple update scheme, in which posterior expectations ${\overset{˜}{\mu }}^{\left(i\right)}$ are driven by a mixture of prediction errors ${\overset{˜}{\varepsilon }}^{\left(i\right)}$ that are defined by the equations of the generative model.

In neural network terms, Eq. (A.3) says that error-units compute the difference between expectations at one level and predictions from the level above (where $\xi^{\left(i\right)}$ are precision weighted prediction errors at the *i*th level of the hierarchy). Conversely, posterior expectations are driven by prediction errors from the same level and the level below. These constitute bottom–up and lateral messages that drive posterior expectations towards a better prediction to reduce the prediction error in the level below. In neurobiological implementations of this scheme, the sources of bottom–up prediction errors are generally thought to be superficial pyramidal cells, because they send forward (ascending) connections to higher cortical areas. Conversely, predictions are thought to be conveyed from deep pyramidal cells by backward (descending) connections, to target the superficial pyramidal cells encoding prediction error (Bastos et al., 2012; Mumford, 1992).

Note that the precisions depend on the expected hidden causes and states. We have proposed that this dependency mediates attention (Feldman and Friston, 2010). Eq. (A.3) tells us that the (state-dependent) precisions modulate the responses of prediction error units to their presynaptic inputs. This suggests something intuitive – attention is mediated by activity-dependent modulation of the synaptic gain of principal cells that convey sensory information (prediction error) from one cortical level to the next. This translates into a top–down control of synaptic gain in principal (superficial pyramidal) cells and fits comfortably with the modulatory effects of top–down connections in cortical hierarchies that have been associated with attention and action selection.
